# Fear of movement/(Re)injury in low back pain: confirmatory validation of a German version of the Tampa Scale for Kinesiophobia

**DOI:** 10.1186/1471-2474-15-280

**Published:** 2014-08-19

**Authors:** Adina Carmen Rusu, Nina Kreddig, Dirk Hallner, Janina Hülsebusch, Monika I Hasenbring

**Affiliations:** Department of Medical Psychology and Medical Sociology, Faculty of Medicine, Ruhr-University Bochum, Universitätsstr 150, Bochum, 44780 Germany; Department of Psychology, Royal Holloway, University of London, Egham, Surrey UK

**Keywords:** Fear of pain, Fear of (re)injury, Fear of movement, Validation, TSK, Tampa scale for Kinesiophobia, Assessment

## Abstract

**Background:**

The Tampa Scale for Kinesiophobia (TSK), an instrument for measuring fear of movement/(re)injury, has been confirmed as an important predictor for the persistence of pain-related disability. The aims of this study were to evaluate the psychometric properties of a German version of the TSK (TSK-GV), examining aspects of content validity with special focus on fear-avoidance and endurance, and to confirm criterion-related validity in patients with low back pain (LBP).

**Methods:**

A total of 191 patients with LBP were included in this study. Several models with different factor structures from published studies were compared in a confirmatory factor analysis. Internal consistencies of the TSK-GV and its subscales were examined, and correlations with related self-report measures were calculated.

**Results:**

The internal consistency of the TSK-GV was α = 0.73. A two-factor model with 11 items was found to be the best fit for our data. The two factors were labelled *Somatic Focus* (SF) and *Activity Avoidance* (AA). The total score, SF and AA revealed moderate to high correlations with other fear-avoidance variables.

**Conclusions:**

The TSK-GV is a reliable and valid measure for assessing the fear of movement/(re)injury.

**Electronic supplementary material:**

The online version of this article (doi:10.1186/1471-2474-15-280) contains supplementary material, which is available to authorized users.

## Background

Pain-related fear has been shown to be a valid predictor of chronic pain and disability [1, 2]. Additionally, “kinesiophobia” refers to “an excessive, irrational and debilitating fear of physical movement and activity that results from a feeling of vulnerability in regard to a painful injury or reinjury” [3] and is conceptualized as a fear of movement/(re)injury by Vlaeyen et al. [4]. According to cognitive-behavioral models, such as the fear-avoidance models [4, 5] or the avoidance-endurance model of chronic pain [6], painful experiences will elicit a fear of movement/(re)injury in certain individuals, which often leads to behavioral avoidance and, in the long run, disuse, depression and increased disability. Other individuals will respond to pain with cognitions of minimization and/or thought suppression, and endurance behavior accompanied with low levels of pain-related fear and avoidance [7].

The Tampa Scale for Kinesiophobia (TSK) [3] was developed in order to assess fear of movement/(re)injury. The psychometric properties of the TSK have been tested in different languages [5, 8–12] and for different pain disorders (e.g., CLBP [5, 13], osteoarthritis [14], fibromyalgia [15], and neck pain [16]). Construct validity has been demonstrated using measures of disability and other fear of pain questionnaires [5, 17, 18].

There are several versions of the TSK available, with 17, 13, 11 and 4 items [19], respectively. The 17-item version includes 4 inversely coded items, which are not included in most previous studies of the TSK due to their low factor loadings. Studies with all 17 TSK items arrive at different factor solutions with one (Miller et al., 1991, unpublished), three [13] or five [9] factors. Studies with the 13-item TSK usually arrive at a two-factor structure [20–24], although the distribution of the items on the factors varies. The 11-item TSK consistently reveals a two-factor structure, and the items that are used, as well as the item distribution, are invariable across studies [16, 25]. A 12-item version showed a four-factor structure [5], and a 4-item version revealed a one factor structure [15]. Due to the many models that are currently in use, it is important for future research to examine the existing models for their accuracy and usefulness. Previous studies that used a confirmatory factor analysis to compare Vlaeyen’s four-factor model (12 items), Clark’s two-factor solution (13 items) and two one-factor models (13 and 17 items) showed that the two-factor model by Clark et al. [20] provided the best fit [14, 25, 13], which was further found to be invariant across different patient groups (e.g., chronic low back pain and fibromyalgia [23, 24]). In a 2007 study, Roelofs [16] presented a new two-factor structure that was based on the TSK-11 by Woby et al., 2005 [25]. This factor structure also proved to be invariant across pain diagnoses and countries [16, 26].

Because a German version of the TSK has not been available until now, the main objectives of this study were threefold: First, several models from previous studies were examined in a confirmatory factor analysis in order to determine the best fit. Second, the psychometric properties of a German version of the TSK were examined in a sample of patients with low back pain. Finally, construct validity was further investigated by exploring the relation to cognitive-affective and behavioral avoidance, as well as to endurance variables. Criterion-related validity was explored with respect to pain intensity, disability and general distress.

## Methods

### Participants and procedure

A total of 205 adult patients with low back pain were consecutively recruited from participating orthopedic practices from February 2001 until March 2002. Inclusion criteria were: age above 18 years and low back pain that lasted for at least 14 days. Exclusion criteria were: severe injuries of the back (e.g., neoplasms, fractures, and herniated discs, which required immediate surgery), major psychiatric illness, and an insufficient knowledge of the German language. Data from 8 patients could not be evaluated due to missing values. Six patients did not participate because they fulfilled at least one of the exclusion criteria (4 due to herniated discs and neurological findings that indicated surgery and 2 due to neoplasms and an inflammatory disease). Finally, data from 191 patients were available. This sample size fulfilled the criteria for conducting a factor analysis [27]. Prior to participation, patients gave their written informed consent. The study protocol was approved by the Medical Ethics Committee of the Ruhr-University of Bochum. Self-report data were obtained by a personal computer based self-report instrument, which included a detailed medical history, demographic variables and several psychometric and pain-related questionnaires. First, patients underwent a standardized orthopedic examination and, thereafter, completed all of the questionnaires in a fixed sequence during a single appointment. The paper was written in adherence to the guidelines of the STROBE statement (for the full checklist, see Additional file 1).

### Measures

#### Sociodemographic and pain history variables

Patients’ gender, age, marital status, educational level, and medical history, including their number of sick days, were assessed with a general demographic and pain history checklist. The pain questionnaire contained detailed questions about several aspects of pain (e.g., pain intensity, current duration, off-work-days, former surgeries). According to Jensen et al.’s recommendations [28], pain intensity was assessed by a numerical rating scale (NRS) that ranged from 0 (‘no pain at all’) to 10 (‘extremely painful’). Severity of pain was assessed with the Chronic Pain Grade (CPG) [29], which measures Grade 1 (low disability, low pain intensity), Grade 2 (low disability, high pain intensity), Grade 3 (high disability, moderate limitation) and Grade 4 (high disability, severe limitation). Cronbach’s alpha for the German version was 0.82 [30].

#### Fear of movement/(re)injury

The original TSK [3] is a 17-item self-report measure of fear of movement and (re)injury (4-point Likert Scale; 1 = ‘strongly disagree’, 4 = ‘strongly agree’). Four of these items are negatively worded and score inversely (item 4, 8, 12, and 16). Most studies of the TSK chose to eliminate these four inversed items, which resulted in a TSK with 13 items. An 11-item version of the TSK has also shown promising psychometric characteristics [16, 25, 26, 31]. For the present study, the original 17 items of the English version of the TSK (including the modification of item order by Vlaeyen et al., 1995 [5]) were translated by forward-backward translation, with consideration to face and content validity [32, 33].

#### Pain anxiety

Anxiety behaviors that are related to pain were assessed with the German version of the Pain Anxiety Symptom Scale (PASS-DE) [34], a 40-item, self-report measure. The original PASS version showed an adequate internal consistency and a considerable degree of validity [35–38]. The German version of the PASS has been shown to be reliable and valid [39], with an internal consistency of α = .94 and a test-retest reliability of α = .84.

#### Depression

Depression was assessed with the Beck Depression Inventory (BDI) [40]. The BDI is a 21-item, self-report measure of depression. It assesses the incidence of various symptoms of depression. It has demonstrated excellent reliability and validity, as well as the ability to discriminate between depressed and non-depressed patients [41, 42]. In the present study, the German version from Kammer (1983) was employed, which has shown high reliability and validity (α = .82) [43].

#### Functional disability

Functional disability was measured with the Pain Disability Index (PDI) [44] and the Oswestry Disability Index (ODI) [45, 46]. Both questionnaires ask the respondent to rate the degree to which pain interferes with their functioning in different areas of daily life. The PDI addresses family/home responsibilities, recreation, social activity, occupation, sexual behavior, self-care and life-support activities. The German PDI is valid and reliable (α = .88) [47]. The ODI concentrates on the following aspects: pain intensity, personal care, lifting, walking, sitting, standing, sleeping, sex life, social life and travelling. The German ODI shows high internal consistency (α = .90) and high test-retest reliability (*r* = .96) [48].

#### Fear-avoidance and endurance-related responses to pain

Fear-avoidance- and endurance-related responses to pain, as well as pain coping strategies, were assessed by the Kiel Pain Inventory (KPI) [49]. The KPI is a self-report instrument that assesses cognitive, affective and behavioral responses to pain. It contains several fear-avoidance and endurance scales. In this study, the following fear-avoidance scales were used: *Anxiety/Depression*, *Help-/Hopelessness*, *Catastrophizing Thoughts*, *Avoidance of Social Activities*, and *Avoidance of Physical Activities*. In order to assess endurance-responses, the following endurance scales were used: *Positive Mood despite Pain*, *Thought Suppression*, *Minimization*, and *Behavioral Endurance*. Internal consistency (Cronbach’s alpha) was above .81 for all of the scales except for the *Thought Suppression* scale, which revealed a score of .78 [49]. The whole KPI, with its 19 subscales, was used in a series of cross-sectional and prospective studies that reflected criteria and construct validity [49–54].

The Fear-Avoidance Beliefs Questionnaire (FABQ) [55] focuses on the patient’s beliefs about how physical activity and work affect low back pain. Psychometric properties for the total score are good [56, 57]. The internal consistency (Cronbach’s alpha) is α = 0.85 and α = 0.91, respectively, and a test-retest reliability of r = 0.78, as well as a split-half-reliability of r = 0.87, were found [56, 57]. In the German version, a factor analysis yielded three factors: *Physical Activity*, *Work as a Cause* and *Work Prognosis*.

### Data analysis

A confirmatory factor analysis was chosen in order to determine the best fit for the German version of the TSK in patients with low back pain. A variety of previously supported models were examined, including a four-factor model with 12 items by Vlaeyen et al. (1995) [5], a two-factor model by Roelofs et al. with 11 items (2007) [16], a two-factor model by Clark with 13 items (1996) [20], a one-factor model with all 17 items (including the inverse items) and a one-factor model that included 13 items (without the inverse items). The analysis was conducted through use of AMOS Graphics. Fit was determined by CMIN, df, GFI, NNFI, CFI and RMSEA and its 90% confidence interval. Missing values in the data set were replaced by means in SPSS. In order to assess the reliability of the final chosen model, internal consistencies (Cronbach’s alpha) were calculated for the factors and the total score of the TSK-GV. In order to examine the validity of the TSK-GV, correlations were calculated between the TSK-GV’s total score and its subscales and disability, depression, pain anxiety, and fear-avoidance beliefs, as well as fear-avoidance and endurance responses and pain intensity and duration. The alpha levels were Bonferroni-corrected. Correlations with depression and disability were considered in terms of concurrent criterion validity, whereas correlations with pain anxiety, catastrophizing and fear-avoidance beliefs regarding physical activity were examined for convergent construct validity.

## Results

### Demographic characteristics

Of the 191 patients, thirteen were on a pension, with two patients receiving temporary pension and two patients having applied for a pension. Nine patients had received surgeries in the last three months. Demographic characteristics did not differ from those that were found in chronic pain populations [58]. Table 1 reports the descriptive findings, including sociodemographic, pain and disability variables.

**Table 1:** **Descriptive statistics of the study sample (N = 191)**

	Mean (SD)
Age (years)	50.1 (11.3)
Duration of pain (years)	6.2 (8.5)
Number of consultations	17.8 (21.3)
Actual pain intensity (0–10)	3.2 (2.2)
Average pain intensity during the last 7 days (0–10)	4.3 (2.0)
ODI (0–10)	1.6 (1.1)
PDI (0–10)	2.9 (1.8)
	**N/%**
Female	105/55,0%
On sick leave	26/13,6%
Days off work	12.24 (48.77)
Von Korff chronic pain grade (CPG)	
– Grade 1	55/28,8%
– Grade 2	50/26,2%
– Grade 3	49/25,6%
– Grade 4	37/19,4%

ODI: Oswestry Disability Index, PDI: Pain Disability Index.

### Confirmatory factor analysis

Five models were compared through use of a confirmatory factor analysis. The four-factor model by Vlaeyen et al. (1995) [5] and the two-factor model by Roelofs et al. (2007) [16] showed the best fit (see Table 2). The two one-factor models each showed bad fit, while the one-factor model with the inverse items showed markedly worse goodness-of-fit than the one-factor model without these items (see Table 2). The model by Clark et al. (1996) [20] and the two one-factor models with and without the inverse items showed inferior fit and were excluded from further analysis.

**Figure 1:**
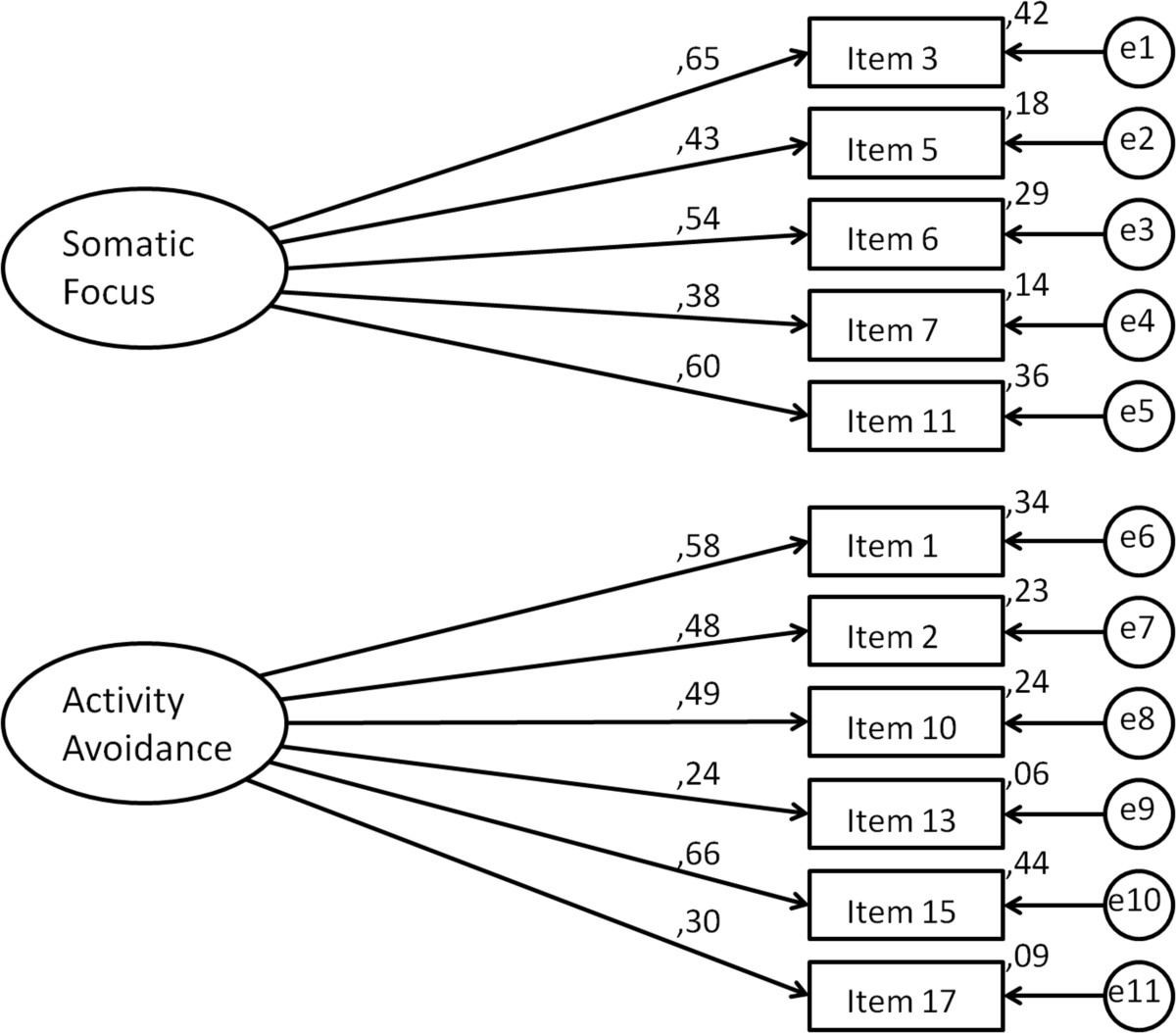
**The TSK-GV's factorial structure and loadings.**

The four-factor model by Vlaeyen (1995) [5] and the two-factor model by Roelofs (2007) [16] were examined for internal consistency regarding both the total score and the subscales. The internal consistency for the two-factor model was marginally better for the total score and superior for the subscales than the internal consistency for the four-factor model and was, thus, chosen for further analysis. Factor loadings for the chosen two-factor model are presented in Figure 1. See the resulting TSK and TSK-GV in English and German in the Additional files 2 and 3.

**Table 2:** **Goodness-of-fit statistics from a confirmatory factor analysis of published TSK models applied to the TSK-GV**

Model	CMIN (df)	GFI	NNFI	CFI	RMSEA (90% CI)
Vlaeyen et al., 1995 [4]	60.45 (48)	.951	.949	.963	.037 (.000-.063)
Roelofs et al., 2007 [16]	61.01 (43)	.946	.915	.933	.047 (.012-.072)
Clark et al., 1996 [20]	101.75 (64)	.924	.867	.891	.056 (.034-.075)
One factor, 13 items*	116.48 (65)	.913	.821	.851	.065 (.045-.083)
One factor, 17 items	288.33 (119)	.848	.598	.648	.087 (1.63-2.16)

*Without inverse items.

### Scoring and Internal consistency

Factor scores were calculated by summing the items and dividing this by the number of items. Descriptive statistics, scale intercorrelations, correlations regarding total score and internal consistencies (Cronbach’s alpha) are shown in Table 3.

**Table 3:** **Reliability and intercorrelations of the TSK-GV total score and its subscales**

Scale	No. of items	Means	SD	Cronbach’s Alpha	Intercorrelations of subscales	
					SF	AA
Factor SF	5	8.44	2.78	0.64		
Factor AA	6	12.92	3.45	0.63	0.423**	
TSK-GV	11	21.34	5.28	0.73	0.804**	0.879**

**Correlation is significant at the 0.01 level (2-tailed), N = 191; SF: Somatic Focus, AA: Activity Avoidance.

### Validity analysis

#### Construct validity

Construct validity was measured by investigating the correlations between the two-factor TSK-GV and its subscales and theoretically related measures, such as measures of pain-related fear-avoidance, endurance and pain anxiety (Table 4). As expected, significant correlations between the TSK-GV total score and pain anxiety, catastrophizing and fear avoidance variables were found. Both of the TSK-GV subscales were significantly correlated with pain anxiety. Especially high positive correlations were found between the subscale SF and *Fearful Thinking* and *Cognitive Anxiety*, while moderate correlations were found between SF and *Physiological Anxiety*, as well as AA and *Fearful Thinking* and *Escape/Avoidance*. SF was correlated with work-related and physical activity-related fear-avoidance beliefs on a low-to-moderate level, while AA only showed a strong correlation with fear-avoidance beliefs due to physical activity. In regard to avoidance and endurance variables, SF showed more consistent correlations with the fear-avoidance subscales of the KPI than AA, which was mainly correlated with *Avoidance of Social Activities* and *Avoidance of Physical Activities*, as well as *Help-/hopelessness*. Fear-related variables, such as *Anxiety/Depression* and *Catastrophizing* (after the Bonferroni correction), only showed significant correlations with SF. With respect to discriminant validity, among the endurance-related variables, *Minimization* was significantly negatively correlated with the total score and both subscales of the TSK-GV, while *Positive Mood despite Pain* showed significant negative correlations with the TSK-GV total score and the SF subscale. *Endurance Behavior* was significantly negatively related to the TSK-GV; however, this correlation disappeared after the Bonferroni correction. *Thought Suppression* was unrelated to the TSK-GV.

**Table 4:** **Correlations between kinesiophobia/(re)injury and fear-avoidance beliefs, pain anxiety, pain coping, disability, depression and pain**

Validity criteria	SF	AA	TSK-GV
PASS-DE	.532**	.415**	.553**
PASS-DE - Fearful thinking	.568**	.422**	.580**
PASS-DE - Cognitive Anxiety	.483**	.272**	.434**
PASS-DE - Escape/Avoidance	.329**	.403**	.444**
PASS-DE - Physiological Anxiety	.400**	.259**	.384**
FABQ – work	.258**	.233**	.286**
FABQ – physical activity	.255**	.469**	.435**
KPI – Fear-avoidance-variables			
Anxiety/Depression	.218**	.114	.191**
Help-/Hopelessness	.383**	.277**	.389**
Catastrophizing	.312**	.167*	.277**
Avoidance of Social Activities	.253**	.203**	.269**
Avoidance of Physical Activities	.198*	.233**	.263**
KPI – Endurance variables			
Positive Mood despite Pain	-.287**	-.171*	-.268**
Thought Suppression	.038	.025	.030
Minimization	-.252**	-.214**	-.282**
Endurance Behavior	-.075	-.181*	-.168*
BDI	.187*	.160*	.206**
ODI	.208**	.205**	.240**
PDI	.280**	.186*	.269**
Average pain intensity (last week)	.074	-.009	.030
Duration of current pain	.021	-.008	-.008

**Correlation is significant at the 0.01 level (2-tailed); *Correlation is significant at the 0.05 level (2-tailed). Bivariate correlations that remain significant after a Bonferroni adjustment (p < .005) are printed in bold. TSK: Tampa Scale for Kinesiophobia; TSK-GV: German version of the TSK; SF: Somatic Focus, AA: Activity Avoidance; PASS: Pain Anxiety Symptoms Scale; BDI: Beck Depression Inventory; PDI: Pain Disability Index; ODI: Oswestry Disability Index; KPI: Kiel Pain Inventory; FABQ: Fear-Avoidance Beliefs Questionnaire.

#### Criterion validity

Criterion validity was measured by examining the correlations between the TSK-GV total score and its subscales and measures of depression and disability. Disability and depression showed significant correlations with the TSK-GV’s total score. Disability showed a more consistent correlation with SF than with AA. Depression was correlated with the total score, but it was not correlated with the subscales (after the Bonferroni correction). Average pain intensity and pain duration were unrelated to the total score and the subscales.

## Discussion

The present study evaluated the factor structure, the internal consistency and the validity of a German version of the TSK (TSK-GV) using a sample of patients with low back pain (LBP). The inverse items were eliminated from the TSK-GV after determining that they were detrimental to a good fit. Important issues refer to certain new aspects of validation with respect to behavioral avoidance and pain-related endurance variables, such as *Positive Mood despite Pain*, *Thought Suppression*, and *Behavioral Endurance*.

Between the models that were examined in the present study, the four-factor model by Vlaeyen et al. (1995) [5] and the two-factor model by Roelofs et al. (2007) [16] emerged as the models with the best fit in the confirmatory factor analysis. An examination of the reliability of the TSK-GV that was built after each model showed that the two-factor solution by Roelofs et al. (2007) [16] produced better results. Therefore, the two-factor solution was chosen as the final model for the TSK-GV. This 11-item TSK-GV model also seems to be the economically sound decision because it is the shortest reliable possibility and, therefore, reduces the patients’ burden. It is also supported by previous studies [16, 31, 59].

In the present study, adequate levels of internal consistency were found for the TSK-GV total score (α = 0.73). The subscales SF and AA showed internal consistency values that were slightly below the desired value of .70, with SS’s alpha = .64 and AA’s alpha = .63. Shorter scales that have less than 10 items are still adequate with an alpha above .60 [60], and French et al. (2007) [13] state that they see the reduced subscale reliabilities that they also found in their study with the TSK-13 as a reflection of the small number of items on the scales rather than of problems with the items per se. Nevertheless, the reliability results indicate that it could be adequate to use the TSK-GV’s total score in clinical practice, especially because the total score shows better reliability than the subscales. Unlike French et al. (2007) [13], the present study did not find a very high subscale intercorrelation (see Table 3), which suggests that the factors in the 11-item version of the TSK measure rather distinct concepts within the main concept of fear of (re)injury. Both subscales showed very high correlations with the total score. These results support the subscales as valid parts of the main concept of fear of (re)injury.

Another emphasis of the present study was on the thorough examination of the validity of the TSK-GV total score and the separate factors. According to prior studies [4, 13, 20, 24], fear of movement/(re)injury was expected to positively relate to depression, catastrophizing, pain anxiety and fear-avoidance beliefs regarding physical activity and disability. Higher TSK-GV total scores were indeed significantly correlated with higher levels of general pain anxiety, fear-avoidance beliefs and emotional, cognitive and behavioral fear-avoidance responses, such as help-/hopelessness, catastrophizing and avoidance of social and physical activities. Activity-related fear-avoidance beliefs showed a stronger correlation with the total score than the work-related ones, which is consistent with French et al. (2007) [13]. The TSK-GV total score was negatively associated with measures of endurance responses, such as *Positive Mood despite Pain* and *Minimization*, while *Endurance Behavior* and *Thought Suppression* were not significantly related. In general, the correlation pattern was consistent with the construct “kinesiophobia” and with different cognitive-behavioral models of pain [4–6]. Positive correlations between several fear-avoidance variables, general distress and the TSK-GV support the assumptions of a pathway from pain, cognitions of catastrophizing and/or help-/hopelessness via pain-related fear of movement/(re)injury to behavioral avoidance. Negative correlations between the TSK-GV and *Positive Mood despite Pain* and between the TSK-GV and cognitions of *Minimization* support one of the endurance pathways, which suggests that low levels of pain-related fear and avoidance are accompanied by high eustress-endurance [6, 7].

Concerning the first TSK-GV subscale *Somatic Focus* (SF), we found a correlation pattern that mainly matched the one for the TSK-GV total score. The subscale *Activity Avoidance* (AA) differed from this pattern slightly, as it was mostly related to pain anxiety, activity-related fear-avoidance beliefs, help-/hopelessness, avoidance of social and physical activities and negatively to minimization. The correlations between the TSK-GV’s AA subscale and the avoidance subscales of the FABQ and the KPI, as well as the negative correlations between the AA subscale and the KPI endurance scales, support the validity of AA being a measure of avoidance. The low or insignificant correlations with endurance are consistent with previous research: Tkachuck and Harris (2012) [59] found low but significant negative correlations between the TSK-11’s AA subscale and measures of physical functioning (stair climb and sit-stand). AA was also able to uniquely predict performance in these tasks of physical functioning. Because avoidance measures are scarce, AA may be useful in this regard. In sum, the high correlations with pain anxiety, the moderately positive correlations with avoidance and fear-avoidance beliefs and the moderately negative correlations with certain aspects of pain-related endurance support the construct validity of the TSK-GV.

In accordance with previous findings [13, 20], positive correlations between the TSK-GV total score and pain-related disability and depression were found, supporting criterion related validity. The SF subscale again showed the same correlation pattern as the TSK-GV total score. For the AA subscale, only a significant correlation with disability that was measured by the ODI was found. Disability that was measured by the PDI and depression did not remain significantly correlated with the TSK-GV after a Bonferroni correction.

Regarding the pain variables, significant correlations between the TSK-GV total score and its subscales and pain duration or pain intensity could not be observed. This finding is in line with previous research [20], while other studies [5, 13] found that the TSK scores were positively related to self-report of clinical pain. Vlaeyen (1995) [5] did not find pain intensity to predict fear of (re)injury and concluded that fear of (re)injury most likely occurs independently of current pain intensity. This suggestion supports the notion of Crombez et al. (1999) [17], who proposed that the expectation of pain may be more debilitating than the actual pain. This could indicate that the extent of kinesiophobia in patients with low back pain is independent of the duration and average pain intensity of the current pain that they are experiencing.

### Limitations

The results of the current study are limited to patients with low back pain. The study was part of a large multicenter study about back pain; therefore, the sample was limited to patients with back pain. Because the data are cross-sectional, conclusions of cause and effect cannot be drawn. The reliability that is stated in this study only refers to internal consistency, as repeated measures for test-retest reliability were not included.

## Conclusions

The results of the present study indicate good psychometric properties of the German version of the TSK (TSK-GV) in low back pain patients. The psychometric properties of the TSK-GV are comparable to the Dutch and English versions. The TSK-GV demonstrates an acceptable level of internal consistency and good construct and criterion-related validity. The present findings support the fit of a two-factor model that is identical to the one supported by Roelofs et al. (2007) [16]. In future studies concerning the TSK-GV, the consistency of the model in other groups of patients should also be explored. The psychometric characteristics of the TSK-GV should be examined more thoroughly by means of test-retest reliability and measures of sensitivity. Instead of the comprehensive Kiel Pain Inventory, the KPI-derived short version Avoidance-Endurance Questionnaire (AEQ) [61] may be used when assessing fear-avoidance and endurance-related pain responses on cognitive, affective and behavioral levels. In addition to the self-report data that were used in the present study, objective behavioral measures should also be observed. Longitudinal studies are needed to clarify the causality between pain, disability and pain-related fear-avoidance and endurance [7].

## Electronic supplementary material

Additional file 1:**STROBE Statement—Checklist of items that should be included in reports of** ***cross-sectional studies.***(PDF 142 KB)

Additional file 2:The German Tampa Scale for Kinesiophobia. / Die deutsche Tampa Scale for Kinesiophobia (TSK-GV). (PDF 20 KB)

Additional file 3:The Tampa Scale for Kinesiophobia (TSK).(PDF 75 KB)

Below are the links to the authors’ original submitted files for images.

Authors’ original file for figure 1

## Abbreviations

TSK: Tampa scale for Kinesiophobia
TSK-GV: German version of the TSK
LBP: Low back pain
SF: Somatic focus
AA: Activity avoidance
CPG: Chronic pain grade
PASS: Pain anxiety symptoms scale
BDI: Beck depression inventory
PDI: Pain disability index
ODI: Oswestry disability index
KPI: Kiel pain inventory
FABQ: Fear-avoidance beliefs questionnaire
CFA: Confirmatory factor analysis.
