# Supplementary material for: Fear of movement/(Re)injury in low back pain: confirmatory validation of a German version of the Tampa Scale for Kinesiophobia
Source: BMC Musculoskelet Disord. 2014 Aug 19;15:280. doi: 10.1186/1471-2474-15-280 (PMC4246485; doi:10.1186/1471-2474-15-280)
Supplement: Supplementary file 2 — Additional file 2:The German Tampa Scale for Kinesiophobia. / Die deutsche Tampa Scale for Kinesiophobia (TSK-GV). (PDF 20 KB) [file 12891_2013_2314_MOESM2_ESM.pdf]

Tampa Scale for Kinesiophobia - German Version (TSK-GV).

Mit den nachfolgenden Fragen möchten wir untersuchen, wie Sie selbst zu Ihren Schmerzen stehen.

Bitte geben Sie an, in welchem Maß Sie der vorgegebenen Aussage zustimmen. Bitte kreuzen Sie dafür bei jeder Frage das entsprechende Kästchen an. (Ein Kreuz pro Frage.)

Item	Frage	Überhaupt **nicht** einverstanden	Mehr oder weniger **nicht** einverstanden	Mehr oder weniger einverstanden	Völlig einverstanden
**1**	Ich habe Angst davor, dass ich mich möglicherweise verletze, wenn ich Sport treibe.	☐	☐	☐	☐
**2**	Wenn ich versuchen würde, mich über die Schmerzen hinweg zu setzen, würden sie noch schlimmer.	☐	☐	☐	☐
**3**	Mein Körper sagt mir, dass ich etwas sehr Schlimmes habe.	☐	☐	☐	☐
**4**	Mein Gesundheitszustand wird von anderen nicht ernst genug genommen.	☐	☐	☐	☐
**5**	Wegen des Schmerzproblems ist mein Körper für den Rest meines Lebens gefährdet.	☐	☐	☐	☐
**6**	Schmerz bedeutet immer, dass ich mich verletzt habe.	☐	☐	☐	☐
**7**	Die sicherste Art, zu verhindern, dass meine Schmerzen schlimmer werden, ist einfach darauf zu achten, dass ich keine unnötigen Bewegungen mache.	☐	☐	☐	☐
**8**	Ich hätte nicht so viel Schmerzen, wenn nicht etwas Bedenkliches in meinem Körper vor sich ginge.	☐	☐	☐	☐
**9**	Meine Schmerzen sagen mir, wann ich mit dem Training aufhören muss, um mich nicht zu verletzen.	☐	☐	☐	☐
**10**	Ich kann nicht all die Dinge tun, die gesunde Menschen machen, da ich mich zu leicht verletzen könnte.	☐	☐	☐	☐
**11**	Niemand sollte Sport treiben müssen, wenn er/sie Schmerzen hat.	☐	☐	☐	☐

© TSK-GV. Rusu, A.C.; Kreddig, N.; Hallner, D.; Hülsebusch, J.; Hasenbring, M.I. (2014).
