# Supplementary material for: Fear of movement/(Re)injury in low back pain: confirmatory validation of a German version of the Tampa Scale for Kinesiophobia
Source: BMC Musculoskelet Disord. 2014 Aug 19;15:280. doi: 10.1186/1471-2474-15-280 (PMC4246485; doi:10.1186/1471-2474-15-280)
Supplement: Supplementary file 3 — Additional file 3:The Tampa Scale for Kinesiophobia (TSK).(PDF 75 KB) [file 12891_2013_2314_MOESM3_ESM.pdf]

Additional file 3. The Tampa Scale for Kinesiophobia (TSK).

With the following questions, we would like to examine how you see your pain.

Indicate your degree of agreement with the following statements. Please mark the according box after each statement with a cross (one cross per statement).

Item	Statement	Strongly **dis**agree	**Dis**agree	Agree	Strongly agree
**1**	I'm afraid that I might injure myself if I exercise.	☐	☐	☐	☐
**2**	If I were to try to overcome it, my pain would increase.	☐	☐	☐	☐
**3**	My body is telling me I have something dangerously wrong.	☐	☐	☐	☐
**4**	People aren't taking my medical condition seriously enough.	☐	☐	☐	☐
**5**	My accident has put my body at risk for the rest of my life.	☐	☐	☐	☐
**6**	Pain always means I have injured my body.	☐	☐	☐	☐
**7**	Simply being careful that I do not make any unnecessary movements is the safest thing I can do to prevent my pain from worsening.	☐	☐	☐	☐
**8**	I wouldn't have this much pain if there weren't something potentially dangerous going on in my body.	☐	☐	☐	☐
**9**	Pain lets me know when to stop exercising so that I don't injure myself.	☐	☐	☐	☐
**10**	I can't do all the things normal people do because it's too easy for me to get injured.	☐	☐	☐	☐
**11**	No one should have to exercise when he/she is in pain.	☐	☐	☐	☐

TSK.
