# Supplementary figures and images for: Fear of movement/(Re)injury in low back pain: confirmatory validation of a German version of the Tampa Scale for Kinesiophobia
Source: BMC Musculoskelet Disord. 2014 Aug 19;15:280. doi: 10.1186/1471-2474-15-280 (PMC4246485; doi:10.1186/1471-2474-15-280)

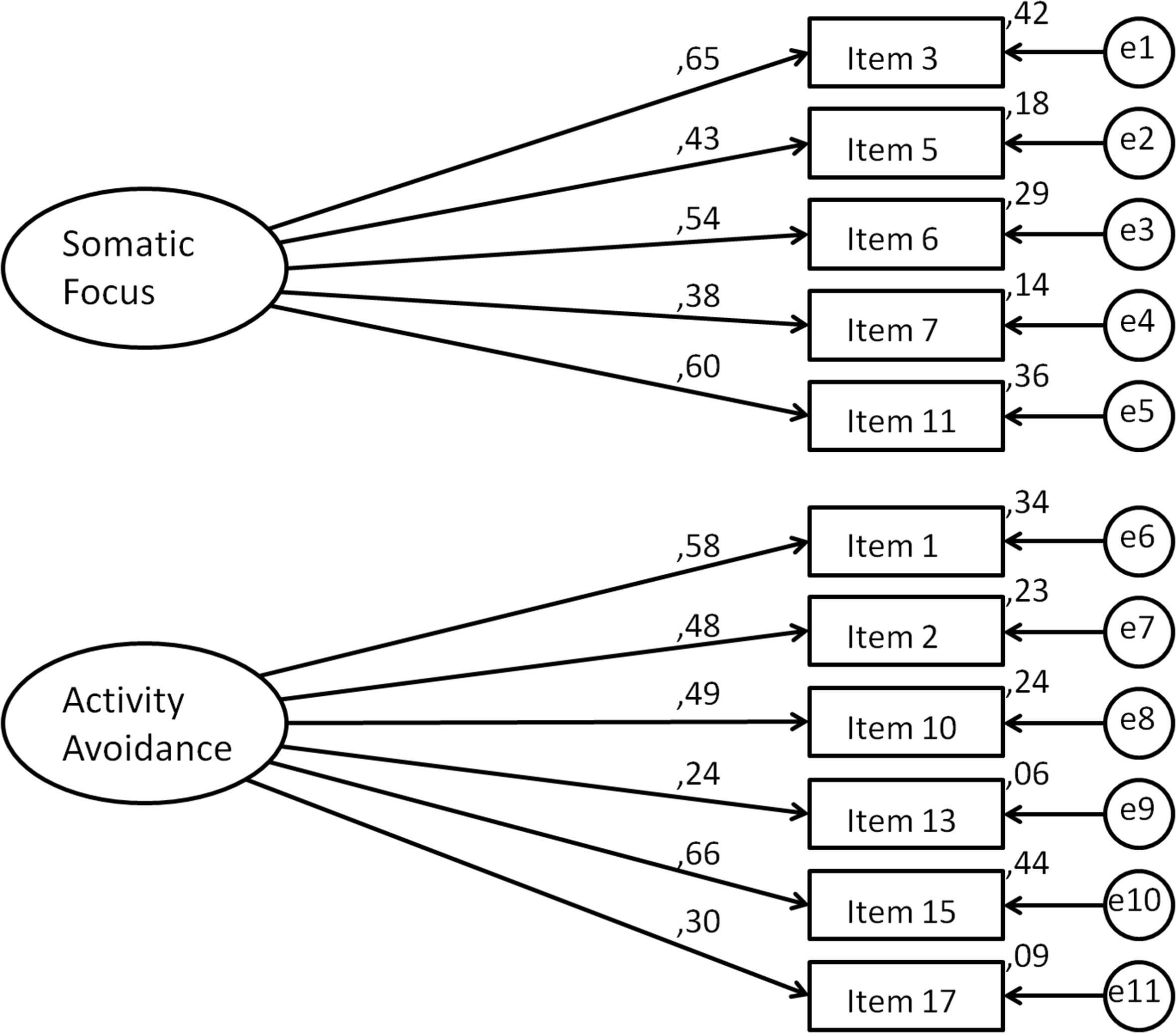

Supplement: Supplementary file 4 — Authors’ original file for figure 1 [file 12891_2013_2314_MOESM4_ESM.tif]
